# Illicit drug use and violence in acute psychosis among acute adult admissions at a South African psychiatric hospital

**DOI:** 10.4314/ahs.v18i1.17

**Published:** 2018-03

**Authors:** Robert Wicomb, Lyndall Jacobs, Naasika Ebrahim, Megan Rensburg, Muiruri Macharia

**Affiliations:** 1 Department of Psychiatry, Faculty of Medicine and Health Sciences, Stellenbosch University, Cape Town South Africa; 2 Acute Admissions Unit, Lentegeur Psychiatric Hospital, 7786 Cape Town, South Africa; 3 Division of Chemical Pathology, Faculty of Medicine and Health Sciences, Stellenbosch University, Cape Town South Africa

**Keywords:** Illicit drug use, violence, acute psychosis, psychiatric hospital

## Abstract

**Background and objective:**

The prevalence of mental illness and illicit substance use has increased markedly in South Africa's Western Cape Province, over the last 2 decades; potentially increasing demand for psychiatric care. This paper describes the demographic and substance use profile of patients admitted to Lentegeur (LGH), the largest of the four psychiatric hospitals in the Province.

**Methods:**

Medical records, patient interviews and other clinical notes were used to collect data on demographics, illicit substance use, violent behaviour and utilization of rehabilitative services for patients (n=535) admitted to LGH between 1 August 2012 and 31 January 2013.

**Results:**

Majority of admissions were male (65.6%) and younger (69.8% < 35 years) compared to females (62.6% >35 years). Overall, 255 (49%) used an illicit substance, (24% females and 63% males). Majority of substance users were youth (18–35 years) in both males (83.1%) and females (73.8%). Cannabis and methamphetamine were the most popular drugs in males (56.3% and 34.9%) and females (17.9% and 16.2%) with the highest rates being among the youth. Violence was common among both men (60.7%) and women (40.8%); among the violent, 67% of males and 35.6% of female used substances. Only 5.5% of drug users utilized formal drug rehabilitation services.

**Conclusion:**

Substance use and violence were high, yet only a small proportion of the patients utilised available drug rehabilitation services. This may have implications on psychotic relapses, morbidity and subsequent pressure on financial resources within the health care system. Efforts are needed to maximise utilisation of existing rehabilitative resources for these patients.

## Introduction

The Western Cape Province has the highest provincial 12-month and lifetime prevalence of mental disorders in South Africa^1^. The province's burden of mental disorders is further compounded by the confluence of psychosocial problems such as illicit drug use and gang /community violence, which place an increased demand on psychiatric services^2^–^3^. Psychiatric patients using illicit substances, when compared to those who do not, often require more behavioural management, have more readmissions, involve costlier (comorbid) treatment and are less likely to comply to follow up at community treatment centres^4^–^6^. Membership of gangs, very prevalent in this province, is also associated with increased levels of mental illness and use of health services^7^.

There are suggestions that, in line with global patterns, illicit drug use and drug-related risk-taking have increased in post-apartheid South Africa^8^. But besides a recent study linking methamphetamine (colloquially known as Tik) use to increased psychiatric admissions in Western Cape 3, data on how substance use impacts psychiatric admissions is sparse and largely anecdotal. The aim of this study was to describe the burden of acute psychosis requiring legal certification at Lentegeur Psychiatric Hospital over a 6-month period, in terms of the rate of admissions to the Acute Adult Inpatient Admissions Unit, as well as its associated descriptive factors; in particular, violence and substance abuse.

## Methods

### Setting

Lentegeur Hospital is the tertiary drainage centre for district and regional hospitals located in the three major regions of Mitchell's Plain sub-urban district (MPH), the Khayelitsha sub-urban district (KDH), and the rural regions of Helderberg, Overberg and Worcester/Winelands. These regions comprise a total population of around 2 million people^9^. The Mitchell's Plain district is characterised by high rates of gangsterism, substance abuse and crime. Over 90% of the region's population is of mixed ancestry, also known as “Coloured”. Like Mitchell's Plain, Khayelitsha is a product of the previous regime's racial segregationist policies (Apartheid) and comprises an almost exclusively (99%) black African population, living mostly inside informal dwellings and characterised by grinding poverty. The rural drainage area of Cape Winelands, Overberg and Heidelberg displays more heterogeneity in ethnicity, socio-economic status, housing, and education status.

### Data collection and analysis

This study was a retrospective audit of all patients (18 – 60 years of age) admitted as acutely psychotic under the Mental Health Care Act of South Africa 2002 (for involuntary or assisted care), between 1^st^ August 2012 and 31^st^ January 2013 to the Acute-Adult Admissions Unit of LGH in Cape Town. The study was based entirely on medical records and therefore involved no more than minimal risk to the patients or adverse implications on their rights and welfare. The records included clinical interviews with the patients, collateral information gained from the family, as well as clinical notes from the referring district hospital. The Ethics Committees of Stellenbosch University and Lentegeur Hospital approved the study and waived informed consent and the latter additionally approved access to patients' records. We ensured that patient personal and identifiable data were anonymized and/or stored in secure, password-protected storage. Incentives were neither given to participants nor was there any opportunity to offer them given the non-contact retrospective medical record review design.

Data was obtained from the medical records in the patient hospital folders, which included clinical interviews with the patients, collateral information gained from the family, as well as clinical notes from the referring district hospital. This data included demographic information (age, gender, and drainage area), illicit drug use, violent behavior, and any prior utilization of formal drug counseling and rehabilitation services. Violent behavior was defined as a physical assault to self or others (staff, other patients, family) and/or damage to property, from the time of onset of symptoms of the current episode of acute psychosis, up to and including the current admission. Substance use was defined as the ingestion or inhalation, or injection, of illicit drugs (methamphetamine (Tik), cannabis, methaqualone (Mandrax), crack cocaine, or heroin) as reported by either the patient, or caregiver, or if noted as observed/positively tested in the documentation from the referral hospital. This definition was restricted to the use of illicit substances clinically deemed to be related to the onset and maintenance of the current episode. Data was summarized descriptively using Microsoft Excel.

## Results

### Demographics

A total of 535 patients were admitted to the unit during the 6-month study period of whom 15 (2.8%) were excluded as readmissions. The final sample of 520 patients (Table 1) was predominantly male (65.6%) and originated nearly evenly from KDH (34.6%); MPH (35.0%) and rural (30.4%). Male admissions were predominantly young (69.8% < 35 years) compared to females, a majority of whom (62.6%) were older than 35 years.

**Table 1:** Substance use and referral origin of the entire sample according to gender and age

	Total	Drainage			Violence	Sud	Cannabis	Tik	Heroin	Other	Ldts

		KDHL	MPH	RURA							
**Males**											
18–25	120	52	39	29	75	99	92	64	1	25	5
26–35	118	58	38	22	70	78	67	45	1	22	3
36–45	62	24	20	18	37	25	23	6	0	5	1
45+	41	6	19	16	25	11	10	4	1	4	0
**Sub Total (%)**	**341 (65.6)**	**140 (41.1)**	**116 (34.0)**	**85 (24.9)**	**207 (60.7)**	**213 (62.5)**	**192 (56.3)**	**119 (34.9)**	**3 (0.9)**	**56 (16.4)**	**9 (2.6)**
**Females**											
18–25	24	6	8	10	13	14	12	12	0	1	0
26–35	43	9	17	17	20	17	11	12	0	2	4
36–45	52	8	25	19	18	4	3	4	0	2	1
45+	60	17	16	27	22	7	6	1	1	0	0
**Sub Total**	**179 (34.4)**	**40 (22.3)**	**66 (36.9)**	**73 (40.8)**	**73 (40.8)**	**42 (23.5)**	**32 (17.9)**	**29 (16.2)**	**1 (0.6)**	**5 (2.8)**	**5 (2.8)**

**TOTAL (%)**	**520**	**180 (34.6)**	**182 (35.0)**	**158 (30.4)**	**280 (53.8)**	**255 (49.0)**	**224 (43.1)**	**148 (28.5)**	**4 (0.8)**	**61 (11.7)**	**14 (2.7)**

### Substance use, rehabilitation and violence

**Figure 1:**
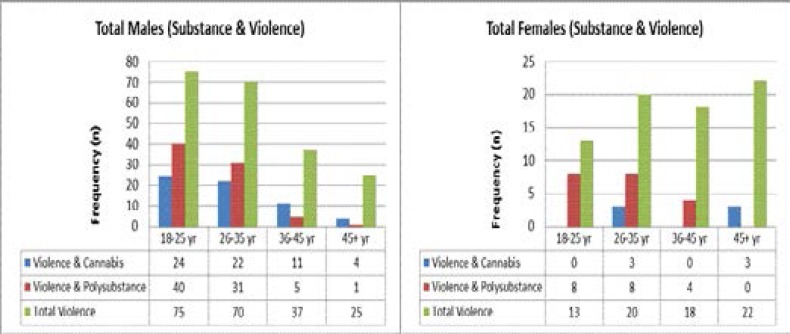
Substance use and violence in male and female admissions

A total of 255 (49%) patients admitted to using an illicit substance, constituting 24% of total females and 63% of total males (Table 1). The largest proportion of the substance users were youth (18–35 years) in both males (83.1%) and females (73.8%). Overall illicit drug use decreased with age among males but remained relatively stable among females, albeit at much lower rates (Fig. 1). Cannabis and methamphetamine/tik were the most popular drugs in both males (56.3% and 34.9%) and females (17.9% and 16.2%) with the highest rates again being among the youth group (Table 1). Other illicit drugs, including heroin, methaqualone, ecstasy and cocaine, were used to a much lesser extent and were almost exclusively limited to males. Only a small proportion of male (4.2%) and female (11.9%) drug users (overall 5.5%) utilized formal drug rehabilitation services. For the latter, nearly all were in the 26–35 age-group (Table 1).

Violence was quite common among both male (60.7%) and female (40.8%) admissions (Table 1). Table 2 further examines the relationship between violence and illicit drug use. Two out of every three (67%) violent males indicated using illicit drugs compared to about one in every three females (36%). Rates were highest among the MPH cohort, whilst KDH had the greatest gender difference.

**Table 2:** Violence and drug use according to drainage area

Variable n (%)	OVERALL	KDH	MPH	RURAL
**Violent**				
Males	207 (60.7)	91 (65)	67 (57.8)	49 (57.6)
Females	73 (40.8)	15 (37.5)	33 (50.0)	25 (34.2)
**Substance**				
Males	213 (62.5)	92 (65.7)	72 (50.0)	49 (57.6)
Females	42 (23.5)	6 (15.0)	20 (30.3)	16 (21.9)
**Violent < using drugs**				
**Males**	138 (66.7)	60 (65.2)	49 (73.1)	29 (59.2)
**Females**	26 (35.6)	2 (13.3)	15 (45.5)	9 (36.0)

## Discussion

This study describes adult patients admitted to the acute wards of a South African psychiatric hospital between August 2012 and January 2013. The results confirm anecdotal observations regarding increased rates of admissions, illicit drug use and violence. We found half of the proportion of the patients admitted in the study period were violent and/or used illicit drugs, yet only a very small proportion utilised available drug rehabilitation services.

There were 535 admissions during this 6-month study period, which is a 22% (n=438) increase compared to the corresponding period of August 2008 to January 2009 (not shown in this study), and thus clearly resonates with concerns that demand for mental health care is rising without corresponding upgrades in existing infrastructure and personnel. During both periods, most of the admitted patients were male, although their proportion was much higher in the latter phase. Male admissions were predominantly young whilst females were mostly mature adults. The reasons for this pattern are unclear but we speculate it partly relates to a possible later onset of psychiatric illness in women, or it may be a reflection of social factors, such as access to services and a higher societal tolerance for disturbed behaviour among females. The latter may be supported by a preponderance of male admissions in our sample.

The results indicate a high rate of substance use among the LGH acute admissions. Men, especially youth, were more likely than females to use substances. This supports previous South African data^8^. The results possibly reflect the perception that men, particularly the youth, are more naturally inclined to risk taking^10^–^11^ whilst drug use may be less in women as the degree of shame and stigma associated with the activity is much greater for women because of gender-based stereotypes that hold them to different standards^12^. Consistent with past data, cannabis and methamphetamine were the most popular illicit substances.

Violence during the psychotic episode was also very prevalent and, as with substance use, particularly high in the MPH cohort — probably a reflection of the high occurrence ofpoverty, crime and gangsterism in that region mentioned earlier. Whilst the level of poverty in the Khayelitsha region is even greater, levels of comorbid violence and substance abuse among the predominantly black African populace seems to be comparatively lower and are comparable to the rural regions. Nonetheless, the extent of methamphetamine use among patients in Khayelitsha was higher than anticipated as the drug is not considered popular in Black African communities^8^.

We noted a relation between comorbid violence and illicit substance use, especially among the youth, where illicit drugs use was very high in violent patients. Also, most substance users tended to be violent. The nature of this study, however, precludes any comment of causal linkages. In mature and elder patients, the proportion of violent females was high, even though the incidence of illicit substance use was low. It is unclear whether this pointed to a delay in admission of female clients.

Although a well-resourced drug rehabilitation infrastructure is available within the metropolitan region, the rate of utilization by the patients was disappointingly low, which likely contributes to high rates of psychotic relapses in our population. The reason for the dismal rate of utilisation is unclear, but we speculate it relates to several factors, including ineffective awareness measures and poor accessibility for the target community. A vicious cycle develops starting with a relapse of substance use, leading to violence and psychoses, resulting in worsened inpatient morbidity, and consequently demanding higher levels of care that depletes financial resources and erodes mental health staff morale, leading to staff burnout and impacting on the quality of service delivery.

The bulk of the data was retrospectively collected and is therefore limited by the degree of accuracy of the original source. Similar limitation exists on substance use data where reliance was almost solely on self-report data, as only a handful had urine tests done at the referral centres.

## Conclusion

The study provides a picture of a burgeoning psychiatric patient population at LGH which is increasingly prone to violence and substance misuse. The sub-optimal utilization of drug rehabilitation is likely to aggravate the burden on - and thus the attrition of - experienced professional staff. We hope this study will encourage a coordinated and integrated approach for better service delivery, and the utilisation of preventive and rehabilitative substance use resources in the communities as well as within hospital environments.

## References

[1] Herman AA, Stein DJ, Seedat S, Heeringa SG, Moomal H, Williams DR (2009). The South African Stress and Health (SASH) study: 12-month and lifetime prevalence of common mental disorders. S Afr Med J.

[2] Dada S, Plüddemann A, Parry C, Erasmus J, Bhana A, Burnhams NH (2012). Monitoring alcohol and drug abuse trends in South Africa (July 1996 – December 2011). SACENDU Research Brief.

[3] Vos PJ, Cloete KJ, le Roux A, Kidd M, Jordaan GP (2010). A retrospectivereview of trends and clinicalcharacteristics of methamphetamine-related acute psychiatric admissions in a South African context. Afr J Psychiatry.

[4] Breslow RE, Klinger BI, Erickson BJ (1996). Acute intoxication and substance abuse among patients presenting to a psychiatric emergency service. Gen Hosp Psychiatry.

[5] Curran GM, Sullivan G, Williams K, Han X, Collins K, Keys J (2003). Emergency department use of persons with comorbid psychiatric and substance abuse disorders. Ann Emerg Med.

[6] Te Wildt BT, Andreis C, Auffahrt I, Tettenborn C, Kropp S, Ohlmeier M (2006). Alcohol related conditions represent a major psychiatric problem in emergency departments. Emerg Med J.

[7] Coid JW, Ullrich S, Keers R, Bebbington P, DeStavola BL, Kallis C (2013). Gang membership, violence, and psychiatric morbidity. Am J Psychiatry.

[8] Peltzer K, Ramlagan S, Johnson BD, Phaswana-Mafuya N (2010). Illicit drug use and treatment in South Africa: a review. Subst Use Misuse.

[9] Statistics South Africa (2011). Census 2011; Statistical release P0301.4.

[10] Arnett JJ (1992). Reckless behaviour in adolescence: A developmental perspective. Dev Rev.

[11] Harris CR, Jenkins M, Glaser D (2006). Gender differences in risk assessment: why do women take fewer risks than men?. Judgm Decis Mak.

[12] O'Brien P (2001). Making it in the free world: Women in transition from prison.

